# Is pubertal timing associated with involvement in bullying in middle adolescence?

**DOI:** 10.1080/21642850.2014.881259

**Published:** 2014-02-05

**Authors:** Eveliina Jormanainen, Sari Fröjd, Mauri Marttunen, Riittakerttu Kaltiala-Heino

**Affiliations:** ^a^ School of Medicine, University of Tampere, Tampere, Finland; ^b^ School of Public Health, University of Tampere, Tampere, Finland; ^c^ National Institute for Health and Welfare, Helsinki, Finland; ^d^ Faculty of Medicine, University of Helsinki, Helsinki, Finland; ^e^ Department of Adolescent Psychiatry, Helsinki University Hospital, Helsinki, Finland; ^f^ Tampere University Hospital, Tampere, Finland

**Keywords:** bullying, pubertal timing, adolescence, mental health, population study

## Abstract

Off-time pubertal maturation is associated with mental disorders, presumably due to stress caused by deviation from peers that could also attract negative attention and result in being bullied. Stress related to off-time maturation could be acted out by involving oneself in bullying behavior. The associations between pubertal timing and involvement in bullying have so far not been a focus of research. The objective is to explore associations between off-time pubertal maturation and involvement in bullying. Cross-sectional and longitudinal associations between self-reported pubertal timing and involvement in bullying as victims and perpetrators were studied in a sample of 2070 Finnish adolescents aged 15–17 who participated in the Adolescent Mental Health Cohort Study. Internalizing (depression) and externalizing (conduct disorder) symptoms were controlled for. The adolescents were recruited to response the baseline survey in 2002–2003 (T1) and follow-up two years later (T2). In T1, response rate was 94.4% of all eligible students. The 2070 participants in T2 comprised 63.1% of the T1 participants. Early maturation among boys was cross-sectionally associated with being a bully, and late maturation with exclusion from peer group. After two years, the associations had disappeared. Among girls, no associations were detected between pubertal timing and involvement in bullying. Off-time pubertal maturation places boys at risk for involvement in bullying. The influence is transient and supports the stressful change hypothesis of pubertal timing.

## Introduction

Puberty is an important time of development and growth in the life of an individual. It is also a very sensitive period psychologically, and mental health problems that start in puberty often continue to adulthood (Costello, Mustillo, Erkanli, Keeler, & Angold, 2003). Adolescents feel it is important to fit in with the group, have same age friends and to feel accepted. The timing of puberty varies from 8 years in early maturing girls to 15 years in late maturing boys. When the average age, for example, of menarche is 12–13 years, early and late maturing adolescents differ significantly from their age group. Physical and psychological changes may be bewildering, impair self-esteem and increase stress. As a consequence, a development which differs significantly from that of peers can be a danger to the mental health of the adolescent (Graber, Lewinsohn, Seeley, & Brooks-Gunn, 1997; Kaltiala-Heino, Koivisto, Marttunen, & Fröjd, 2011). Early maturing adolescents do not have age mates in the same developmental phase as they themselves are, and therefore cannot find support or share their feelings with others. Late maturers on the other hand may experience concern about their late development. Attention received from adults and peers may be unwelcome and exacerbate stress and problems. Early maturing girls have been reported to suffer more depression, early sexual behavior and alcohol abuse than others (Angold, Costello, & Worthman, 1998; Copeland et al., 2010; Graber et al., 1997; Kaltiala-Heino, Marttunen, Rantanen, & Rimpelä, 2003; Weichold, Silbereisen, & Schmitt-Rodermund, 2003). The results for boys have not been as consistent. Depending on the study, either late or early puberty has been associated with mental health problems (Graber et al., 1997; Kaltiala-Heino et al., 2003).

Bullying is a serious and common problem among young people, and fortunately is nowadays more often addressed. Bullying is often defined as a continuous action intended to harm someone else physically or mentally. It also includes a power-imbalance between the bully and the victim. Bullying is most commonly divided into three subgroups: physical (hitting, kicking and pushing), verbal (namecalling, spreading rumors) and social (leaving someone alone against their wishes, exclusion from social interaction). Cyperbullying is bullying that takes place using information technologies like social media, email and mobile phones (Lindfors, Kaltiala-Heino, & Rimpelä, 2012). Physical bullying is more common among boys, while social bullying is more common among girls. Verbal bullying is equally common in both sexes, cyberbullying again seems more common among the girls (Kumpulainen et al., 1998; Lindfors et al., 2012). Boys are usually bullies and victims more frequently than girls. Bullying is most common in early adolescence between the ages of 12 and 13, when approximately 10–20% of youngsters are estimated to be involved in it (Kaltiala-Heino & Fröjd, 2011; Olweus, 1993). As adolescents grow older, bullying is observed to decline (Kumpulainen, Räsänen, & Henttonen, 1999).

An association between bullying and mental health problems has been reported in numerous studies. The link between them is undeniable, but the nature of their temporal relationship is not as clear, and varies between studies. Mental health problems may cause bullying and victimization, but being a bully or a victim may also cause mental health problems (Kaltiala-Heino & Fröjd, 2011; Kaltiala-Heino, Rimpelä, & Rantanen, 2000; Kumpulainen et al., 1999). Being involved in bullying has been associated with a wide range of symptoms; psychosomatic symptoms, depression, low self-esteem, social phobia, behavioral problems, suicidal ideation and attempts, drug abuse, eating disorders and even psychotic experiences (Heikkilä et al., 2013; Kaltiala-Heino, Fröjd, & Marttunen, 2010; Kaltiala-Heino et al., 2011; Kaltiala-Heino et al., 2000; Klomek et al., 2008; Liang, Flisher, & Lombard, 2007; Scholte, Engels, Overbeek, de Kemp, & Haselager, 2007). Of these, depression has been studied most. The relationship between depression and bullying also varies depending on the country, age of the research participants and study methods. Some studies suggest that depression often precedes bullying (Klomek et al., 2008), while others suggest that depression is an outcome of victimization (Bond, Carlin, Thomas, Rubin, & Patton, 2001; Salmon, James, & Smith, 1998), or that the association may be different in boys and in girls (Kaltiala-Heino et al., 2010). Bullies have also been found to suffer from depression, and bullying could be a way of acting-out (Kaltiala-Heino & Fröjd, 2011; Kaltiala-Heino et al., 2000; Olweus, 1993; Rigby, 2002). Bully victims have been discovered to have the greatest number of mental health problems with bullies and victims in second place (Forero, McLellan, Rissell, & Bauman, 1999; Kaltiala-Heino & Fröjd, 2011; Kaltiala-Heino et al., 2000).

Thus, both being involved in bullying and pubertal timing have been associated with mental health problems. Peer relationships are of outmost importance in adolescent development. It has been suggested that off-time maturing peers develop symptoms due to the stress of facing physical changes without support from peers in a similar situation. However, it may be that the role of peers is even more active in the negative sense. Adolescents maturing earlier or later than their peers may become targets of unpleasant attention and bullying because of being different. They may also themselves develop problem behaviors such as bullying because of feelings of being outsiders or perceiving negative attention. In a Finnish study (Kaltiala-Heino et al., 2003), a link between pubertal timing and involvement in bullying was found while studying the effect of pubertal timing on mental health. In girls very early puberty increased the risk of being a bully over threefold and for boys over twofold compared with peers with late puberty.

The aim of this study was to analyze whether pubertal timing has an effect on involvement in bullying, taking into consideration all aspects of it – bullying, victimization and social isolation. We also assessed whether the possible association differs between boys and girls, and if the association persisted during a follow-up of two years. As mental health problems are most often associated with both pubertal timing and bullying, we analyzed whether internalizing (depressive symptoms) or externalizing (aggression and delinquency) mental health problems explained the associations between pubertal timing and involvement in bullying, or if there was some association independently of these. In light of the existing literature, we expected to find that early maturing girls would be most involved in bullying both as victims and as bullies, and that late maturing boys would report victimization, whereas early maturing boys would report being bullies.

## Materials and methods

### Materials

The material for our study was acquired from the Adolescent Mental Health Cohort Study, a survey study conducted in two Finnish cities, Tampere and Vantaa. The data were collected during the academic year 2002–2003 (T1) and academic year 2004–2005 (T2). As a result, a two-year follow-up was established. For a more detailed description of the methodology, see the relevant papers (Fröjd, Kaltiala-Heino, & Marttunen, 2011; Ritakallio et al., 2008).

The eligible subjects were all adolescents who were enrolled in the ninth grade of the Finnish-speaking comprehensive schools in the two cities and who had turned 15. The initial sample in the first survey (T1) was 3278 pupils of whom 49.1% were girls (*n* = 1609). Their mean age was 15.5 years (sd 0.36). Response rate at T1 was 94.4% of all eligible pupils in the participating schools. The second survey was conducted among the first wave respondents two years later. The 2070 adolescents who also responded to the second survey (T2, response rate 63.1%) were the subjects of the present study. Of these, 56.4% (*n* = 1167) were girls. The mean age of the respondents at T2 was 17.6 years (sd 0.41).

At T1 the questionnaire was given out to all ninth-grade pupils in Vantaa and Tampere. The person-identifiable survey was completed in a school lesson and was supervised by a teacher. For those absent from school, a second chance was given to participate within a couple of weeks. If a pupil was not present on either occasion, the questionnaire was sent home by post. If no response was received after this, it was concluded that the student was not willing to participate. The follow-up survey was sent two years later to all those who answered at T1 and could still be reached. At this point, the adolescents had mostly moved to upper secondary schools or vocational studies. The survey was completed either at school, sent by post or done on the Internet.

### Measures

The study addressed three different areas of bullying: being a bully, being a victim and being left alone against one's wish. Bullying was defined in the study in the following way:

We say a pupil is being bullied when another pupil, or a group of pupils, say or do nasty things to him or her. It is also bullying when a pupil is being teased repeatedly in a way she or he does not like. But it is not bullying when two pupils of about the same strength quarrel or fight. (King, Wold, Tudor-Smith, & Yossi, 1996)

The original response alternatives were “never”, “once or twice”, “two to three times a month”, “about once a week” and “many times a week”. The answers were analyzed as a dichotomized variable, which determined whether the pupil had been a part of bullying or not. Answers “about once a week” and “many times a week” were defined as a yes, whereas answers “never”, “once or twice” and “two to three times a month” were defined as a no (Kaltiala-Heino et al., 2010).

Pubertal timing was elicited from the participants at T1. Pubertal timing was defined as the age of menarche for girls and the age of oigarche for boys. The question on pubertal timing (how old were you when you had your first periods/ejaculations) had seven response alternatives: “I haven't yet had periods/ejaculations”, “10 years or younger”, “11 years”, “12 years”, “13 years”, “14 years” and “15 or more”. The pubertal timing variable was divided into three categories. In this way the variable represented early, on-time and late maturing adolescents. Respondents answering “10 years or younger” and “11 years” were defined as maturing early, “12 years” and “13 years” as on-time and “14 years”, “15 years or older” and “I haven't yet had my periods/ejaculations” as maturing late (Kaltiala-Heino et al., 2011).

The internalizing dimension of mental disorders was in this study represented by depressive symptoms. Depressive symptoms were assessed at T1 and at T2 with a Finnish modification of the Beck Depression Inventory (R-BDI) (Raitasalo, 2007). The Finnish modification of the 13-item BDI has demonstrated good psychometric properties among adolescents (Kaltiala-Heino, Rimpelä, Rantanen, & Laippala, 1999). The 13 questions involving statements on feelings, cognition and physical symptoms related to depression were scored (0–3) and summarized. The theoretical scores range from 0 to 39, 39 indicating the greatest severity. Depressive symptoms were used as a continuous variable in the analysis. In the present data, the R-BDI displayed good internal consistency with a Cronbach's alpha of 0.84.

Externalizing symptoms were assessed with the Aggression and Delinquency Scales of the Youth Self-Report Questionnaire (YSR) (Achenbach, 1991), which consists of 29 questions about behavior and emotional life. Each question had three response options: “never”, “sometimes” and “often”. It was possible to score 0–2 points from each question, and the sum of these scores represented the degree of externalizing symptoms. The theoretical scores thus range from 0 to 58. Externalizing symptoms score was used as a continuous variable. The Cronbach's alpha for the Delinquency Scale was 0.67, and for the Aggression Scale it was 0.86.

### Statistical methods

All analyses were performed separately for girls and boys, as it has been previously noted that the psychological effect of bullying and pubertal timing differs between the sexes. The association between the variables was first examined with cross-tabs analysis and chi-square statistics, which yielded information on the frequency of bullying in the different pubertal timing categories (early, on time and late maturers). Pubertal timing was used as a categorical variable with which to assess the frequency of bullying, victimization and being left alone. This was done for both time points to see if there was a difference at the ages of 15 and 17. In this way we constructed 12 different cross tables.

The bivariate associations between pubertal timing and depressive symptoms/externalizing symptoms and between depressive symptoms/externalizing symptoms and involvement in bullying at ages 15 and 17 were analyzed using one way analysis of variance (ANOVA) and independent samples *t*-test.

To study multivariate associations, logistic regression analyses were used. Being involved in bullying (as a bully, victim or being left alone, each in turn), was used as the dependent variable in the logistic regression model. The categorical pubertal timing was used as an independent variable. The late maturing adolescents were used as the reference category. After carrying out the basic regression, three models were constructed to assess the effect of age, depressive symptoms and externalizing symptoms at T1 on results at T1 and T2. The first model used age and depressive symptoms, the second age and externalizing symptoms, and the third included all three. Chronological age was controlled for in the regression analyses together with externalizing and internalizing symptoms, because even if the participants were very homogenous in age, even small differences at this stage of development could have an impact. Age was calculated from dates of birth and of responding, and is expressed in years and used in the analyses as a continuous variable. The value *p* = 0.05 was used as the threshold value for statistical significance in all analyses. Odds ratios (OR) with 95% confidence intervals (CI) are reported.

In the tables, statistically significant findings are highlighted with bold text.

The analyses were performed using statistical package for social sciences (SPSS) for Windows (20.0) software.

### Drop-out from follow-up

The response rate to the second questionnaire was acceptable. However, altogether 28% of the girls and 48% of the boys responding to the first survey dropped out in the follow-up. Pubertal timing was not an explanatory factor for the drop-out in girls, whereas in boys non-normative pubertal timing was associated with a lower probability of answering the second questionnaire (early, 50%; on time, 59%; late, 49%; *p* = 0.001). Those dropping out reported more often being bullies at T1 than those participating at follow-up (5% vs. 3%, *p* = 0.002). However, drop-outs were not more often subjected to bullying at T1 than participants (4% vs. 3%, *p* = 0.6), nor left alone by peers against their wishes (3% vs. 2%, *p* = 0.6). Depression at T1 was more common in drop-outs (12% vs. 9%, *p* = 0.02). Higher levels of delinquency indicated a lower probability of responding (*p* = 0.001), whereas aggressiveness was not associated with the probability of responding. Higher age was associated with dropping out with 11% vs. 5% being 16 years or older at T1 (*p* = 0.001).

### Ethical considerations

The study was approved by the Ethics Committee of Pirkanmaa Hospital District. The subjects gave written informed consent after being informed about the study and the voluntariness of participation. Their parents were informed in advance by letter, but parental consent was not requested because the Finnish legislation on medical research allows minors from 15 years to consent alone.

## Results

### Distribution of variables studied

Pubertal timing did not differ statistically significantly between the sexes (Table 1). The vast majority of adolescents stated that they had never been victims, bullies or been left alone against their wishes at either time point. Boys statistically significantly bullied others and were victims of bullying more often than girls at the age of 15. Being left alone was equally common in both sexes. By the age of 17 bullying and victimization had declined, but being left alone was at approximately the same level as two years earlier.

**Table 1.** Timing of oigarche/menarche reported at age 15, being involved in bullying at age 15 (T1) and two years later at age 17 (T2) and mean values (standard deviations) of the Depressive and Externalizing Symptom Scales among Finnish adolescents (%).

	Boys T1(age 15) *n* = 903	Boys T2(age 17) *n* = 903	Girls T1(age 15) *n* = 1167	Girls T2(age 17) *n* = 1167
*Timing of oigarche/menarche*				
10 or less	4.8		2.7	
11	12.7		17.0	
12	28.3		35.6	
13	29.2		27.7	
14	13.5		13.3	
15 or later	3.2		2.1	
Has not occurred yet	4.0		1.3	
Missing	4.2		0.3	
*Has been bullied*				
Never	79.1	86	85.2	92.5
Once or twice	14	9.5	10.8	4.7
2–3 times a month	1.9	0.8	1.9	0.4
Once a week	2.3	1.2	1.3	0.3
Many times a week	2.4	1.2	0.9	0.3
Is not at work or studying	–	0.6	–	1.4
Missing	0.3	0.7	0	0.3
*Has bullied others*				
Never	68.8	83.3	86.1	93.2
Once or twice	24.4	12.1	12.1	4.5
2–3 times a month	2.3	1.2	0.3	0.3
Once a week	1.8	1.2	0.8	0.3
Many times a week	2.5	1.1	0.5	0.1
Is not at work or studying	–	0.6	–	1.4
Missing	0.2	0.6	0.2	0.2
*Has been left alone*				
Never	86.2	87.0	84.2	83.7
Once or twice	10.3	7.5	11.1	10.3
2–3 times a month	0.8	1.1	2.1	1.8
Once a week	1.0	0.9	0.9	1.1
Many times a week	1.3	1.8	1.3	1.3
Is not working or studying	–	0.7	–	1.4
Missing	0.4	1.0	0.5	0.4
*Depression (BDI)*				
Mean score (sd)	2.0 (3.5)	1.9 (3.6)	3.1(4.1)	3.0 (4.2)
*Aggression (YSR)*				
Mean score (sd)	7.7 (5.8)	6.3 (5.4)	9.0 (5.0)	7.6 (4.7)
*Delinquency (YSR)*				
Mean score (sd)	5.3 (2.7)	5.4 (2.8)	5.4 (2.7)	5.3 (2.3)

R-BDI mean score and Aggression Scale on YSR was statistically significantly higher for girls at both time points. The delinquency mean scores of YSR were nearly the same for boys and girls at both time points (Table 1).

### Associations between depressive and externalizing symptoms with pubertal timing and involvement in bullying

Both girls and boys displayed statistically significantly more externalizing symptoms at age 15 the earlier their pubertal timing (Table 2). Depressive symptoms at age 15 were not statistically significantly associated with pubertal timing.

**Table 2.** Depressive and externalizing symptoms according to timing of oigarche/menarche among 15-year-old Finnish boys and girls (mean (sd)).

	Boys				Girls			
	11 or less	12–13	14 or more	*p*	11 or less	12–13	14 or more	*p*
Depressivesymptoms^a^	2.08 (3.7)	1.78 (3.0)	2.31 (4.4)	.173	3.44 (4.4)	3.09 (4.0)	2.84 (4.3)	.353
Externalizingsymptoms^b^	15.1 (8.5)	12.7 (7.3)	11.6 (7.7)	.000	16.0 (7.1)	14.3 (6.7)	13.8 (7.3)	<.001

Mean values (standard deviations) of the symptoms scores according to pubertal timing groups were studies using ANOVA.

^a^In the present data, depression scores ranged 0–39.

^b^In the present data, externalizing symptoms scores ranged 0–48.

Victims had statistically significantly higher externalizing symptom scores at age 15 than those not subjected to bullying (male *p* = 0.012, female *p* < 0.001), as did those who were bullies compared with non-bullies (male *p* < 0.001, female *p* < 0.001). Externalizing symptoms at age 15 were not associated with being left alone at T1. Externalizing symptom scores at T1 among boys were statistically significantly associated with being a bully at T2 (*p* < 0.001) and among girls with being a victim at T2 (*p* = 0.022).

Depressive symptoms were statistically significantly more common at T1 among male victims (*p* < 0.001), among bullies (*p* < 0.001) and also among boys who were left alone against their wishes (*p* < 0.001). For girls, depressive symptoms were statistically significantly more common among victims (*p* = 0.002), and among those girls who were left alone against their wishes (*p* < 0.001). Girl bullies did not have higher scores than other girls. At the age of 17, previous depressive symptoms had no effect on boys being victims or bullies. Previous depressive symptoms were associated with being left alone (*p* < 0.001). Those girls having depressive symptoms at age 15 were more likely to be victims (*p* = 0.049), bullies (*p* = 0.011) and be left alone (*p* < 0.001) at age 17.

### Bivariate associations between pubertal timing and involvement in bullying at ages15 and 17

Among boys, being a bully at age 15 was most common among those with early puberty (*p* = 0.012). Boys with late pubertal timing reported at age 15 having been more commonly left alone by peers than those boys with early or normative pubertal timing (*p* = 0.004). Among girls, pubertal timing was not associated with involvement in bullying at age 15 (Table 3).

**Table 3.** Being involved in bullying as a bully, victim or by being left alone among Finnish boys and girls at age 15 (T1), and two years later (T2), according to age at oigarche/menarche.

	Age at menarche/oigarche				
	11 years or less	12–13 years	14 or more years	Altogether	
	%, *n*/*N*	%, *n*/*N*	%, *n*/*N*	%, *n*/*N*	*p*
*Boys T1*					
Bully	8.9 (14/158)	3.1 (16/519)	4.2 (8/189)	4.4 (38/866)	**.012**
Victim	4.4 (7/158)	3.8 (20/520)	6.9 (13/189)	4.6 (40/867)	.242
Left alone	1.9 (3/157)	1.2 (6/519)	5.8 (11/189)	2.3 (20/865)	**.004**
*Boys T2*					
Bully	3.2 (5/156)	2.0 (10/512)	2.6 (5/189)	2.3 (20/857)	.634
Victim	3.8 (6/156)	1.8 (9/511)	3.2 (6/189)	2.5 (21/856)	.274
Left alone	2.6 (4/156)	2.0 (10/508)	4.3 (8/188)	2.6 (22/852)	.255
*Girls T1*					
Bully	3.0 (7/231)	1.2 (9/738)	1.0 (2/202)	1.5 (18/1171)	.137
Victim	2.6 (6/231)	2.4 (18/739)	2.0 (4/202)	2.4 (28/1172)	.908
Left alone	1.3 (3/230)	2.4 (18/736)	3.0 (6/201)	2.3 (27/1167)	.487
*Girls T2*					
Bully	0.9 (2/225)	0.3 (2/732)	0.5 (1/196)	0.4 (5/1153)	.497
Victim	1.3 (3/225)	0.5 (4/731)	0.5 (1/196)	0.7 (8/1152)	.461
Left alone	4.4 (10/225)	1.9 (14/729)	2.6 (5/196)	2.5 (29/1150)	.120

%, *n*/*N*: differences between groups were studied using cross tabulations with chi-square statistics.

Involvement in bullying at age 17 was not statistically significantly associated with pubertal timing in either boys or girls.

### Multivariate associations between pubertal timing and involvement in bullying at ages15 and 17

Early pubertal timing continued to be statistically significantly associated with bullying others at age 15 among boys after controlling for age and depressive symptoms (*p* = 0.049), but adding externalizing symptoms into the model leveled out the association. Depressive and externalizing symptoms had independent associations with being a bully at age 15 among boys in the final model (depressive *p* = 0.051, externalizing *p* < 0.001) (Table 4). In the multivariate models being subjected to bullying at age 15 among boys was not associated with pubertal timing, but depressive symptoms emerged as predicting victimization (*p* < 0.001). Being left alone was more common among late maturing boys (*p* = 0.002), and depressive symptoms also predicted being left alone (*p* < 0.001).

**Table 4.** Age adjusted OR (95% CI) for involvement in bullying at age 15 by pubertal timing among Finnish adolescent girls and boys.

	Being a bully			Being a victim			Being left alone		
	Model 1	Model 2	Model 3	Model 1	Model 2	Model 3	Model 1	Model 2	Model 3
	Pubertal timing, age and depressive symptoms	Pubertal timing, age and externalizing symptoms	Pubertal timing, age and depressive and externalizing symptoms	Pubertal timing, age and depressive symptoms	Pubertal timing, age and externalizing symptoms	Pubertal timing, age and depressive and externalizing symptoms	Pubertal timing, age and depressive symptoms	Pubertal timing, age and externalizing symptoms	Pubertal timing, age and depressive and externalizing symptoms
**Girls**									
*Pubertal timing*									
Late	Ref	Ref	Ref	Ref	Ref	Ref	Ref	Ref	Ref
On-time	1.2 (0.3–5.8)	1.4 (0.3–7.0)	1.4 (0.3–6.8)	1.3 (0.4–4.0)	1.3 (0.4–4.0)	1.3 (0.4–4.0)	0.9 (0.3–2.6)	0.8 (0.3–2.2)	1.0 (0.4–2.7)
Early	3.0 (0.6–14.5)	2.3 (0.4–12.3)	2.3 (0.4–12.2)	1.3 (0.4–4.9)	1.1 (0.3–4.0)	1.1 (0.3–4.1)	0.4 (0.1–1.9)	0.4 (0.1–1.8)	0.5 (0.1–2.0)
*Depressive symptoms*	**1.1 (1.0–1.2)**	–	1.0 (0.9–1.1)	**1.1 (1.0–1.2)**	–	**1.1 (1.0–1.2)**	**1.2 (1.1–1.2)**	–	**1.2 (1.1–1.2)**
*Externalizing symptoms*	–	**1.2 (1.1–1.3)**	**1.2 (1.1–1.3**)	–	**1.1 (1.1–1.2)**	**1.1 (1.0–1.2)**	–	1.0 (0.9–1.0)	1.0 (0.9–1.0)
**Boys**									
*Pubertal timing*									
Late	Ref	Ref	Ref	Ref	Ref	Ref	Ref	Ref	Ref
On-time	0.9 (0.4–2.2)	0.6 (0.3–1.8)	0.8 (0.3–2.3)	0.6 (0.3–1.3)	0.5 (0.2–1.0)	0.6 (0.3–1.3)	**0.2 (0.1–0.5)**	**0.1 (0.1–0.5)**	**0.2 (0.1–0.5**)
Early	2.6 (1.0–6.8)	1.5 (0.6–4.2)	1.9 (0.6–5.4)	0.6 (0.2–1.7)	0.5 (0.2–1.4)	0.6 (0.2–1.8)	0.3 (0.1–1.2)	**0.3 (0.1–1.0)**	0.3 (0.1–1.3)
*Depressive symptoms*	**1.2 (1.1–1.2)**	–	**1.1 (1.0–1.2)**	**1.2 (1.1–1.2)**	–	**1.2 (1.1–1.3)**	**1.2 (1.1–1.3)**	–	**1.2 (1.1–1.3)**
*Externalizing symptoms*	–	**1.2 (1.1–1.2)**	**1.1 (1.1–1.2)**	–	1.0 (1.0–1.1)	1.0 (1.0–1.0)	–	1.0 (1.0–1.1)	1.0 (0.9–1.0)

Logistic regression analyses are presented entering being a bully, being a victim and being left alone at age 15 as dependent variables.

No association was found in the multivariate analyses between pubertal timing and being a bully, victim or being left alone among 15-year-old girls. Externalizing symptoms were found to be associated with being a bully also after controlling for age and depressive symptoms (*p* < 0.001). In a similar model, both depressive and externalizing symptoms in girls were found to be independently associated with being a victim (depressive *p* = 0.037, externalizing *p* < 0.001). Being left alone was not associated with externalizing symptoms, but both depressive symptoms and chronological age were predictors of being left alone (depressive *p* < 0.001, age *p* = 0.017) (Table 4).

At the age of 17 among boys, early puberty was no longer associated with being a bully. Pubertal timing was not associated with any kind of involvement in bullying at T2. Earlier depressive symptoms seemed to be associated with being a bully among boys (*p* = 0.023), but when externalizing symptoms were added into the model, the association leveled out. Externalizing symptoms on the other hand were independently associated with being a bully (*p* < 0.001). Depressive symptoms at the age of 15 were associated with being a victim and being left alone at age 17 (victim *p* = 0.038, left alone *p* < 0.001) (Table 5).

**Table 5.** Age adjusted OR (95% CI) for involvement in bullying at age 17 by pubertal timing among Finnish adolescent girls and boys.

	Being a bully			Being a victim			Being left alone		
	Model 1	Model 2	Model 3	Model 1	Model 2	Model 3	Model 1	Model 2	Model 3
	Pubertal timing, age and depressive symptoms	Pubertal timing, age and externalizing symptoms	Pubertal timing, age and depressive and externalizing symptoms	Pubertal timing, age and depressive symptoms	Pubertal timing, age and externalizing symptoms	Pubertal timing, age and depressive and externalizing symptoms	Pubertal timing, age and depressive symptoms	Pubertal timing, age and externalizing symptoms	Pubertal timing, age and depressive and externalizing symptoms
**Girls**									
*Pubertal timing*									
Late	Ref	Ref	Ref	Ref	Ref	Ref	Ref	Ref	Ref
On-time	0.6 (0.0–6.3)	0.5 (0.0–5.8)	0.5 (0.0–5.9)	1.0 (0.1–9.2)	1.0 (0.1–8.9)	1.0 (0.1–8.7)	0.7 (0.3–2.1)	0.7 (0.3–2.0)	0.7 (0.3–2.1)
Early	1.7 (0.2–19.8)	1.4 (0.1–16.4)	1.5 (0.1–17.3)	2.4 (0.2–23.1)	2.0 (0.2–20.1)	2.0 (0.2–19.9)	1.7 (0.5–5.0)	1.6 (0.5–4.9)	1.7 (0.5–5.1)
*Depressive symptoms*	**1.1 (1.0–1.3)**	–	**1.1 (1.0–1.3)**	**1.1 (1.0–1.2)**	–	1.0 (0.9–1.2)	**1.1 (1.1–1.2)**	–	**1.2 (1.1–1.2)**
*Externalizing symptoms*	–	**1.1 (1.0–1.2)**	**1.1 (1.0–1.2)**	–	**1.1 (1.0–1.2)**	**1.1 (1.0–1.2)**	–	1.0 (1.0–1.1)	1.0 (0.9–1.1)
**Boys**									
*Pubertal timing*									
Late	Ref	Ref	Ref	Ref	Ref	Ref	Ref	Ref	Ref
On-time	0.8 (0.3–2.5)	0.7 (0.2–2.2)	0.6 (0.2–1.7)	0.6 (0.2–1.9)	0.5 (0.2–1.5)	0.6 (0.2–1.8)	0.5 (0.2–1.3)	0.4 (0.2–1.1)	0.5 (0.2–1.3)
Early	1.3 (0.4–4.8)	0.8 (0.2–3.1)	0.7 (0.2–2.9)	1.4 (0.4–4.5)	1.0 (0.3–3.7)	1.3 (0.4–4.3)	0.7 (0.2–2.4)	0.5 (0.2–1.8)	0.7 (0.2–2.5)
*Depressive symptoms*	**1.1 (1.0–1.2)**	–	1.0 (0.9–1.1)	**1.1 (1.0–1.2)**	–	**1.1 (1.0–1.2)**	**1.2 (1.1–1.3)**	–	**1.2 (1.1–1.3)**
*Externalizing symptoms*	–	**1.1 (1.1–1.2)**	**1.1 (1.1–1.2)**	–	1.0 (1.0–1.1)	1.0 (1.0–1.1)	–	1.0 (1.0–1.1)	1.0 (0.9–1.0)

Logistic regression analyses are presented entering being a bully, being a victim and being left alone at age 17 as dependent variables.

Following the results at age 15, pubertal timing was not associated with being involved in bullying among girls at the age of 17. Previous depressive or externalizing symptoms were not associated with being a bully or being a victim. Previous depressive symptoms on the other hand did predict being left alone at T2 (*p* < 0.001) (Table 5).

## Discussion

Pubertal timing was associated with involvement in bullying among boys at the age of 15. Those maturing early were more likely to be bullies, and those with late pubertal timing were more likely to be left alone against their wishes. Therefore, both maturing early and late was disadvantageous for boys, although in different ways. This is in accordance with earlier research findings concerning the impact of pubertal timing on mental health among boys (Graber et al., 1997; Kaltiala-Heino et al., 2011; Kaltiala-Heino et al., 2003).

A study from Finland found earlier that early maturing boys had more psychiatric problems than those maturing late (Kaltiala-Heino et al., 2003), whereas other studies from the USA have reported that early pubertal timing protects boys against problems and late pubertal timing exposes to them (Graber et al., 1997; Weichold et al., 2003). Early puberty may carry different values among North-American and Finnish adolescent boys. It could be, for example, that in some cultures (like in North America), others, for example parents, teachers and other significant adults, signal to early maturing boys that their maturation is positive and gives them possibilities and responsibility in a way that promotes positive self-perception and hence mental health in the boys. In other cultures (like in Finland), early maturing boys could be assumed more competent than what they actually are and thus left without age adequate support, or could receive the message that they are suspected of heading to trouble, which could then become a self-fulfilling anticipation. These considerations, however, are for the time being speculative and require further research.

When controlling for externalizing symptoms, the association between being a bully and early puberty among 15-year-old boys leveled out and depression had no effect. Externalizing symptoms have previously been reported to be most common among adolescents who bully others, so our findings are not surprising in this respect. Numerous studies have associated being a bully with a greater likelihood of externalizing symptoms. The association seems to be worldwide, as results have been reported from Italy (Menesini, Modena, & Tani, 2009), Sweden (Ivarsson, Broberg, Arvidsson, & Gillberg, 2005), Finland (Kumpulainen et al., 1998), Norway (Undheim & Sund, 2010) and Turkey (Alikasifoglu, Erginoz, Ercan, Uysal, & Albayrak-Kaymak, 2007) to mention a few. It seems that early puberty does not in itself explain involvement in bullying as the perpetrator, but bullying rather seems to be a part of the externalizing symptom dimension. Our finding that being a bully was more common among boys than girls is also consistent with existing research (Forero et al., 1999; Kaltiala-Heino et al., 2003; Kumpulainen et al., 1999; Olweus, 1993; Rigby, 2002; Salmon et al., 1998).

The association between late pubertal timing and being left alone remained significant among boys, even when depressive and externalizing symptoms were controlled for. Rejection from the group was, therefore, not a result from being significantly antisocial or depressed. Later maturation in boys can lead to social problems as these boys are the last ones of their age group to enter puberty (Graber et al., 1997; Weichold et al., 2003), and deviate the most from their peers at age 15. As the impulse given by sex steroids is of utmost importance for brain development in adolescence and psychological development follows neural development (Paus, Keshavan, & Giedd, 2008), it is likely that these boys are mentally younger than their peers, and can be seen as childish or immature and, therefore, be left out.

A novel finding in this study was that even though the timing of puberty did have an effect on being involved in bullying among boys at the age of 15, the association disappeared when the adolescents reached the age of 17. The effect of pubertal timing on bullying was, therefore, transient, and did not persist in later adolescence. Contradictory results have been published on whether the effects of pubertal timing on mental health are transient or persistent (Ge, Conger, & Elder, 2001; Ge et al., 2003; Graber, Seeley, Brooks-Gunn, & Lewinsohn, 2004; Kaltiala-Heino et al., 2011; Taga, Markey, & Friedman, 2006). Earlier studies have suggested that being a bully is quite persistent (Kumpulainen & Räsänen, 2000; Scholte et al., 2007; Sourander, Helstelä, Helenius, & Piha, 2000). This may be the case in general, but the effect of early pubertal timing on bullying gradually declines as adulthood draws nearer. The reasons for this may be many, ranging from acquiring better social skills to becoming less anxious about one's own development, as insecurity is a potential reason for bullying others.

According to the stressful change hypothesis of pubertal timing, stress and vulnerability to problem behaviors and symptoms emerge from being in a phase of change, regardless of when it occurs, and the impact of timing is an artefact that vanishes in the long run when the whole cohort has gone through puberty (Ge et al., 2003; Kaltiala-Heino et al., 2011). Our current results lend support to this theory. The effect of pubertal timing seems to be transient.

Unexpectedly pubertal timing did not have an effect on involvement in bullying among girls. Early maturing girls have been widely observed to have more social and psychological problems than other girls (Copeland et al., 2010; Weichold et al., 2003). We expected that early maturing girls would have been more involved in bullying than other girls, but this was not the case. Early maturing girls displayed more externalizing symptoms than late maturing girls, but they were not more frequently bullies. This suggests that being a bully is among girls a separate phenomenon, not simply a part of externalizing symptom dimension. In the present data, internalizing symptoms were among girls not associated with pubertal timing, which is in contradiction with a number of studies reporting an association between early maturation and internalizing symptoms among girls (for review, see Kaltiala-Heino & Fröjd, 2011). The number of girls involved in all aspects of bullying was significantly lower than in most other studies (Forero et al., 1999; Ivarsson et al., 2005), but in line with recent Finnish studies. Involvement in bullying was even rarer at the age of 17, which was to be expected as the social skills have developed even further, and those maturing late have also caught up with their age peers.

Early pubertal timing was found to be a risk factor for externalizing symptoms in both boys and girls. In earlier studies, early pubertal timing has been linked with both internalizing and externalizing symptoms, especially in girls (Kaltiala-Heino et al., 2003; Kaplowitz, 2004; Weichold et al., 2003), but the data on boys has not been so consistent.

As has been found in earlier studies (Ivarsson et al., 2005; Undheim & Sund, 2010), externalizing symptoms were more common among male bullies, but in contrast to this research, our study found that the victims also had more externalizing symptoms than those peers not involved in bullying. In girls, externalizing symptoms were more common among victims than among others. Depressive symptoms were clearly associated with being involved in any aspect of bullying in boys and girls. The only exceptions were those girls who were bullies, as they did not have more depressive symptoms than others. It has been previously debated whether mental health problems cause bullying (Salmon et al., 1998), or bullying causes mental health problems (Bond et al., 2001) or even if they have any connection at all (Kim, Koh, & Leventhal, 2005). According to our results, externalizing symptoms predict being a bully two years later in boys, but not as clearly in girls. Earlier depressive symptoms are also a risk factor for both genders for reporting being left alone at the age of 17.

Earlier research has been inconclusive as to whether being bullied predisposes adolescents to depression or whether depression is a risk factor for later reporting being bullied. In the present study, depression at age 15 predicted becoming bullied and feeling isolated at age 17 among boys, and feeling isolated at age 17 in girls. This is a noteworthy observation since earlier research on the associations between bullying and mental health has not taken into account pubertal timing and consequent differences in maturation among the subjects.

### Strengths and limitations

This study was based on a large sample of adolescents in the Finnish cities of Tampere and Vantaa. At the age of 15 only few young people are not attending secondary school, as education is compulsory until 16 years of age. The response rate was very good at the first time point (94.4%) and about two-thirds (63.1%) participated in the follow-up, which can be considered acceptable. Those lost to follow-up seemed to have more depression, delinquency and be more likely defined as bullies than those who did answer, but there is no particular reason to assume that this influenced the results (van Loon, Tijhuis, Picavet, Surtees, & Ormel, 2003).

The measures used in this study have previously been used on large studies on adolescents. The Finnish modification of the short 13-item BDI has been widely used to study depression in unselected European populations and in screening depression in clinical work in Finland (Raitasalo, 2007). Self-reported depressive symptoms are important in adolescence, as even without a clinical diagnosis, they commonly cause functional impairment (Lewinsohn, Solomon, Seeley, & Zeiss, 2000). Therefore, in a study like ours, self-report in depressive symptoms is justified. The YSR Scale has been shown in a large Swedish study to be a potential instrument for assessing adolescents' self-reported competencies and problems (Broberg et al., 2001). It can be reasonably assumed that the teenage population of Finland and Sweden resemble each other closely, and thus the use of the YSR is justified.

A possible limitation is that pubertal timing was assessed only by self-reported age at menarche/oigarche. Age at menarche, however, is commonly used and accepted as the explanatory moment to define pubertal age in girls (Rimpelä & Rimpelä, 1993), but there is no equally widely used definition for boys. The age at oigarche is mostly a useful and easy way to define pubertal timing in boys, although not quite as reliable as age at menarche in girls (Carlier & Steeno, 1985). In self-report studies, young boys may be uncertain whether they ejaculate or not, especially when this occurs spontaneously at night. Another method of assessing oigarche would be to measure morning spermaturia (Schaefer, Marr, Seidel, Tilgen, & Schärer, 1990). This technique, however, would require more than a questionnaire, and even after reaching oigarche, sperm cannot be observed in all the urine samples of males. Another way of assessing pubertal timing is to use Tanner stages (Tanner, 1962), but studies using age at menarche/oigarche in this field yield results similar to those obtained using the Tanner stages.

The seemingly small number of adolescents who are bullies may be due to the desire to hide the involvement, as it is commonly known to be disapproved of. Being a victim can also be played down out of shame or misunderstanding. This, however, is unlikely, since the definition of bullying was clearly stated in the questionnaire along with the time definitions. Anonymity was also emphasized, so there was no threat of being exposed. Even though an observational study or peer nomination could give more accurate information on being a victim or a bully than a self-report study, many studies have previously used questions the same or very similar to those used in the present study (Fekkes, Pijpers, & Verloova-Vanhorick, 2004; Forero et al., 1999; Liang et al., 2007).

### Conclusion

Both early and late maturing boys are at risk for involvement in bullying, although in different ways. Early maturing boys bully others most frequently, so their social skills should be taken into account, and their behavioral problems detected as early as possible. On the other hand, with late maturing boys, it is essential to encourage their friendships with peers, as being left alone and lack of friends can be a threat to their mental health. Uncertainty about their physical development, low self-esteem and deviance from their age peers could explain why off-time maturing boys get involved in bullying. The effects of pubertal timing on involvement in bullying are transient.
